# The convergence of financial inclusion across provinces in Vietnam: A novel approach

**DOI:** 10.1371/journal.pone.0256524

**Published:** 2021-08-26

**Authors:** Nhan Thien Nguyen, Ha Son Nguyen, Chi Minh Ho, Duc Hong Vo

**Affiliations:** Research Centre in Business, Economics & Resources, Ho Chi Minh City Open University Vietnam, Ho Chi Minh City, Vietnam; Szechenyi Istvan University: Szechenyi Istvan Egyetem, HUNGARY

## Abstract

Financial inclusion has generally been considered an effective mechanism to support economic growth and reduce Vietnam’s poverty for the last decade. While the importance of financial inclusion to economic growth or macroeconomic stability has been widely examined, it appears that the degree of financial inclusion across Vietnam has not attracted attention from academics and policymakers. In particular, a convergence of financial inclusion across provinces in Vietnam has never been examined. This paper is conducted to examine the static and dynamic distributions of financial inclusion across provinces in Vietnam. The latest three biennial surveys from 2014 to 2018 and a novel approach known as the dynamic kernel density function are used in this study. Our results indicate that Vietnam’s economic growth and development over the 2014–2018 period is relatively inclusive. The evidence also demonstrates that households provided with access to multiple sources of finance depend significantly on the provincial level of income. We also find that provinces located in the national key economic regions, including (i) the Northern region and (ii) the Southern region, appear to achieve a higher degree of financial inclusiveness. Our findings also confirm the catching-up from the financially disadvantaged provinces to financially advantaged provinces locating within the key economic regions. We argue that understanding the asymmetric effect of economic growth on financial inclusion will be helpful for the Vietnamese government in formulating and implementing economic policies promptly to secure the sustainable and inclusive goals of economic growth and development in the future.

## 1. Introduction

Since the economic reform over the last three decades, generally known as “Doi Moi” in 1986, Vietnam has now become a low-middle income country. Vietnam used to be one of the poorest countries on earth. These achievements are mainly thanks to the timely and effortful attempts in economic and political reforms. During this period, the average income of the Vietnamese people has tripled from 3 USD a day in 2002 to 8 USD a day by the end of 2018. The economic development also uplifted 45 million people, which is equivalent to 50 per cent of Vietnam’s population in 2019, out of extreme poverty [1]. Moreover, Vietnam has not experienced an economic recession during the global COVID-19 epidemic.

However, Vietnam’s economy is now transitioning to the new phase of economic growth in which sustainable economic development is a primary concern. Sustainable economic growth is defined by the United Nations (UN) and many non-governmental organizations (NGOs) as economic improvement without being detrimental to future generations. In Vietnam, many socio-economic and environmental issues have emerged as the inevitable consequences of economic growth and unsustainable-caused factors concerning inequality, pollution, urbanization, unequal growth, and competitiveness between sectors and localities. With the focus on asymmetry, the economic disparity between sectors and localities places enormous tension on the urban environment and infrastructure quality. Such inadequacy of the employment opportunities and demand for skilful labour has caused mass movement of people from rural provinces to megacities. According to [2], Vietnam’s urbanization rate has increased from 19.6 per cent with 629 metropolitan areas in 2009 to 36.6 per cent with 802 metropolitan areas in 2016. As of April 2019, the number of urban cities has increased to 830, including two central cities, Hanoi and Ho Chi Minh City, 19 Class-1 urban, 29 Class-2 urban, 45 Class-3 urban, and 80 Class-4 and 655 Class-5 urban. Closely related to urban overpopulation, energy consumption rises also put forward continuity for future economic growth and development. Electricity for residential and industrial uses had been likely tripled over the past ten years. This substantial increase is associated with the constant increase in greenhouse gas emissions of 5 per cent per year [3]. The upcoming middle-income trap challenges these problems. As such, adequate concerns and intervention from policymakers are required.

With the focus solely on the dissimilarity of growth, the gap between regions fails to stash up sufficient capital for growth, which is directly caused by the poor accessibility to the financial market [4–6]. In detail, neoclassical economists consider that the direct driver of growth is the accumulative physical and human capital [7, 8]. These growth factors are typically characterized by vast and non-redeemable investments such as education or capital-intensive manufacturing process, which are apparently unaffordable to agents without hereditary wealth or financial support. In this sense, the well-functioning financial system takes the merit of providing sufficient access to credit-takers, including disadvantaged households and small enterprises. For that reason, policies on universally enhancing financial literacy and depth are key determinants that provide the catching-up effects between socially or geographically disadvantaged and advantaged provinces. We consider Vietnam’s circumstance provides a unique opportunity for this hypothesis to be tested for the following considerations. *First*, despite the constant and fast economic growth, Vietnamese adults who own financial accounts remain unchanged from 2014–2017, which is approximately 31 per cent. Noticeably, that percentage in rural areas has experienced a slight drop from 26 per cent to 25 per cent in the 2014–2017 period [9].

*Second*, the financial outreach strikingly differs from region to region. As an illustration, although the Northern midland and mountain region are geographically adjacent to the Red River Delta region, the proportions of commune or ward with a commercial bank branch are significantly dissimilar (5.3 and 14.3 per cent) [10]. Similarly, the average distance to travel to the nearest bank branch is significantly different from region to region. Households living in the Northern midland and mountain regions must travel proximately 13.9 kilometres (8.7 miles) to receive any form of financial service (cash withdraw, deposit, or loan registration). This distance is only about 4.3 kilometres (or 2.7 miles) in the Red River Delta region. In addition, there are considerable differences in households’ financial behaviour between regions. At the same time, most Northern regions prefer the passbooks, which record the deposits at the banks, as a form of saving than others. We note that over 80 per cent of communes in the Red River Delta region and the Northern midland and mountain regions with residents who possess passbooks. This figure is only 60 per cent in the Mekong Delta region located in the South of Vietnam. People in the Southern regions are also involved in informal credits groups. Sixty per cent of communes with residents participate in informal credit groups [11]. *Third*, acting as the two significant credit-suppliers for rural areas, Agribank (Bank for Agriculture and Rural Development) and BSP (Vietnam’s Bank for Social Policy) are far from meeting the demand for credit in the rural areas. Given approximately 194 different products on offer, the rural banking services are solely for deposit and lending.

With the disparities mentioned above, many governments and banking sectors’ attempts aim to boost financial usage. However, the impact is trivial. Since 2014, the General Statistic of Vietnam has incorporated questions about the households’ current financial usage to keep track of economic growth on the level of financial inclusion. This study is conducted to contribute to a growing body of literature on financial inclusion by non-parametrically investigating the dynamic pattern of households’ financial behaviour in Vietnam. Our literature review indicates that the issue and the approach have not been conducted in previous empirical studies. In this study, evidence for the convergence of financial usage across Vietnamese provinces is presented. Our empirical results indicate that the inter-regional difference of financial usage occurs only within the non-financially focused group. This group mainly include provinces where agriculture activities account for a significant contribution to their provincial gross domestic product. Another significant contribution from this paper is that, for the first time in Vietnam, an index of financial inclusion for each of the 63 provinces across Vietnam is developed and calculated. Finally, based on findings, relevant policy implications have emerged to improve financial inclusion for households, especially those in financially disadvantaged regions.

The structure of this paper is as follows. Following this introduction, section 2 discusses and synthesizes related studies to identify a research gap. Section 3 of the paper presents a research methodology. Empirical findings are then presented and discussed in section 4, followed by section 5 on main conclusions and implications for policy based on the paper’s findings.

## 2. Literature review

Theoretical development of the interaction between growth and finance has circled a rigorous debate between two schools of thought, including the Schumpeterian model and the classical dichotomy. While one school of thought ascertains the essential roles of financial intermediaries and institutional quality on growth [11–13], the other school argues that the functionality of the "real" world is independent of what is called "monetary" factors [14, 15]. However, stylized facts and recent analytic studies on financial depth, resilience and efficacy exhibit a favourable tendency to the former way of thinking. Accordingly, at least four channels through which the financial market influences the future and current aggregate productivity [16–18]. *First*, the quality and quantity of financial services directly influence the wealth distribution and, as such, explain the cross-country growth dissimilarities [19, 20]. In detail, a level of financial accessibility and the initial income level are two main factors that significantly affect the ability of agents to magnify their economic well-being. With restrictive access to financing channels, it is expensive and difficult to invest in human and physical capital to poor households and small enterprises. It is because these investments are often indivisible [21, 22].

Countervailing views of [23, 24] indicate that the effects of initial wealth persist only in the short-run given by the presence of market imperfection. In this sense, their conclusions indicate that countries will eventually reach an identical steady state of growth regardless of their initial capital. With the indivisibility of investments in education or physical capital, [25, 26] attribute the cross-country inconsistency of growth to the pre-determined distribution of wealth and the failure of the lending mechanism. Borrowers have to provide collateral in order to receive a significant reduction of interest rates. As such, high-income countries tend to have an equal distribution of income.

*Second*, financial systems allow agents to put their savings or utilize the investments most efficiently so that none of the growth opportunities in the economy can be missed [13]. If the financial system is under-developed, the “unbanked” or marginalized groups will most likely have to consider levering their wealth, internal resources or even relations to finance the imminent investments against choosing the suboptimal or simply the alternative ones [27]. This mechanism also benefits the supply side of the financial market since it creates channels through which investors can minimally diversify away from the unsystematic risk by holding different types of financial assets with a different date to maturity. Furthermore, access to primary banking services notably reduces the cost of gathering relevant information about firms. These costs are often costly and fixed [28]. Let take the credit information centre or the equity market as an example. Without them, investors have to pay a considerable amount of money to acquire ample pieces of information to make their judgments. However, as [29] articulated, the positive influence of access to finance on growth occurs only in markets with decent contractual enforcement and a well-developed legal system. In short, these authors provide a vivid example of borrowers in Africa countries who apply for loans but have no intention of repaying them. Empirical attempts of [30–32] show that small and micro enterprises encounter many difficulties when approaching financial services since they are not eligible for the requirements on collateral, repayment and administrative costs. These authors also point to the differentials of risk-premium on loans, mainly caused by the asymmetric information between users and suppliers of financial services, which are deleterious to the financial breath, depth and efficiency. Analogously, [33, 34] characterize credit constraints into two forms: credit entry-cost and collateral constraint. The authors also empirically demonstrate that when the credit entry cost is removed, productive firms which previously failed to pay the upfront cost due to insufficient wealth will regain access to finance. Remarkably, they further emphasize that the removal of collateral constraints will greatly facilitate growth.

*Third*, the financial system encourages firms’ specialization and adoption of the advanced manufacturing process [17]. It is often impossible for wealth-constraint or financially excluded firms to employ capital intensive production since the investments are non-convex and permanent. Therefore, small firms will opt to use labour-intensive production lines, which presumably leads to the increasing urbanization and environmental degradation rate and deters growth [35–37]. On the supply side, the financial market significantly reduces the illiquidity of the long-term investments by creating the exit mechanism for investors, who are about to receive the income shock and likely want to convert their illiquid assets to the medium of exchanges [38, 39]. We use the industrial revolution as a typical example. Materials or manufactured products that ignite the first revolution had been invented a long time ago [40, 41]. The financial revolution conditions the new adoption of steamed and waterpower in the manufacturing process in the 18^th^ century and sparks up the emergence of London as the first well-known international financial centre alongside New York [42].

*Fourth*, as discussed in [16, 43, 44], the financial market keeps a watch list of firms whose ex-ante have been securitized or financed. In this sense, a growing body of empirical and theoretical attempts shares consensus that the equity market provides a measurement for managers’ performance after the new project has been monetarily injected by binding the managerial compensation to stock price [45]. It prevents the board of directors from undertaking projects that solely benefit themselves rather than maximize shareholders’ well-being, and therefore adjusts the managerial benefits in line with the owners. Some scholars argue that the more developed the equity market is, the easier it needs to perform hostile takeovers, which can be interpreted as an action that transfers the operating rights from inefficient managers to efficient managers [46, 47]. Findings from empirical studies of [48, 49] show that it is the development of financial intermediaries that determine the cost of estimating the project returns, reduce credit rationing and assure the optimal way of utilizing savings from disparate agents [50]. Attribute the emergence of financial intermediaries to economizing the costs of informational verification [51, 52]. Consider that legal origin and financial development are just catalysts for the relationship between corporate governance and return.

The countervailing arguments [53, 54] indicate that the promotion of financial development does not necessarily economize the information costs inter-and intra- groups of stakeholders, who differ from each other about the views on interests, returns, investment horizons and strategies. As articulated, the information cost exists even between groups of shareholders, who are well-informed or hold the majority of voting rights and who are less informed or hold a small number of voting rights.

The nexus between financial inclusion, growth and inequality has gradually attracted attention from scholars, especially for emerging countries such as Vietnam [55]. Discuss the overlook of economic growth and demographic inequality concerning account ownership across Europe and Central Asia over 2011–2017. Moreover, the authors point to the disparity among adults with financial accounts between these countries in central Asia and the rest of the region. Using the Life-in-Transition survey during the global financial crisis, [56] examine financial inclusion in the forms of deposit accounts and credit accounts. Each of them is jointly conditioned by the function of socio-economic and demographic factors. The results show that the likelihood of one household opting to be financially included is influenced by their current occupancy, type of employment, marital status, education, age, religion, and ethnicity. Focus on the impact of the M-PESA campaign, [57] consider that the increasing access to mobile money has lifted 194,000 Kenyan households out of extreme poverty. Moreover, promoting financial ubiquity also helps people in Kenya increase their savings to 20 per cent.

In sum, it is neither convincing nor practical to deny the contributions of financial development on growth. Financial access is more than a means of exchange since it optimally pools and allocates resources scattered in the economy and avoids extravagance or suboptimal alternatives. Moreover, the financial market applies to control and encourages firms’ engagement in long-term R&D projects. Access to finance also offers financially vulnerable or marginalized groups a helping hand in withstanding idiosyncratic shocks or gives them opportunities to alter their current investment strategies.

## 3. Methodology and data

### 3.1. Data and the construction of household-based financial inclusion index

The Vietnam Household Living Standards Surveys (VHLSS) during the 2014–2018 period is used to calculate the financial inclusion index for 63 provinces in Vietnam. Each VHLSS survey is conducted on a biennial (two-year) basis. It is purported to systematically monitor the changes in living conditions of households in Vietnam, given by the effects of the socio-economic progression. There are approximately 10,000 households across 63 provinces, which are randomly surveyed for their current living conditions and financial needs. With the focus on the financial aspect, eight questions in the eighth section of the survey are asked to demonstrate how respondents are aware of finance. The questions and their measurements are summarized in Table 1 below.

**Table 1:** Questions reflecting how well households are financially literate.

Question type	Question	Measurement
***1. Financial services***		
1.1	Has your household had any saving accounts opened at banks?	Dummy
1.2	Has your household had any passbooks opened at banks?	Dummy
1.3	Has your household had any ATM cards?	Dummy
***2. Lending activities***		
2.1	Has your household had any credit cards?	Dummy
2.2	Has your household borrowed money or goods (including seed, fertilizer) over the last 12 months?	Dummy
***3. Financial market***		
3.1	Has your household has life insurance?	Dummy
3.2	Has your household has non-life insurance?	Dummy
3.3	Has your household has any financial assets in terms of stock, bonds?	Dummy

The answers to these questions only represent the usage dimension of financial inclusion, which represents how well the people of one particular household know about finance. Many factors influence this financial awareness, and the dominant factors are the level of education and the subtlety of financial services [58, 59]. Moreover, financial inclusion is a broad concept, and as such, it requires multidimensional assessments [60, 61]. However, as data is unavailable, in this study, we focus on the key aspect of financial inclusion, which is financial usability.

Utilizing household demographic data, [62] introduce a country-wise index of poverty from three distinct dimensions, each of which stands for three primary needs, including health, education and living conditions. Identically, [63] argue that the selection of six indicators that reflects the current living standards in [62] study should be reduced to three indicators. Moreover, these authors also put forward slight calibrations for how malnutrition, mortality and school enrollment should be [64]. Estimate household financial deprivation by assigning equal weight to four fundamental financial services. These services include the transaction (including checking account), saving (in the forms of debts and equities), credit (in the forms of loans and credit cards), and insurance (including commercial insurance). As such, we use the method of [64] with modifications. Instead of grouping the responses to six questions into four dimensions, we use answers to eight questions in Table 1 with equal weight. Zhang and Posso reduce the significance of each component since some of them are exclusive to others. For example, if households are either using credit cards or loans but not simultaneously, then the weight for credit dimension will be reduced in half. Following saving dimensions, bond and equity markets in Vietnam and other emerging countries are substitutable [20]. Only corporates or governments can issue bonds which is still unfamiliar with a majority of the Vietnamese people. As such, it is unreasonable to attach the use of bonds and stock as a form of saving.

The answers received from the above-surveyed questions are in the form of 0 or 1. As such, we construct our index of financial inclusion for each province by assuming the maximum degree of provincial financial inclusion that a household can achieve is 8 for all questions listed in Table 1. As such, our index of financial inclusion for each province varies from the 0–8 range, in which 0 denotes the “unbanked” or “marginally banked” households, and 8 denotes the highest degree of financial inclusion. The process is repeated for all surveyed households in the sample. Sequentially, we sum up all of these points (scores) by province. We then calculate the ratio between this total sum and the maximum possible point of 8. This ratio is then assigned as the degree of financial inclusion at which each province achieved. As such, our final index of financial inclusion for each province varies within the 0–1 range. More importantly, calculating the above ratio is purported to lessen the heterogeneous effect between provinces caused by regional differences in economic growth levels. The entire process is mathematically expressed as follows.

$$IFI_{jt}=\frac{\sum_{n=1}^{N_{jt}}\sum_{i=1}^{N_{q}}A_{in}}{N_{jt}\times N_{q}}$$

where the *IFI*_*jt*_ denotes the financial usability at province *j* in year *t* (we incorporate three consecutive VHLSS datasets, which is the VHLSS in 2014, 2016 and 2018). *A*_*in*_ represents the answer to the *i*^th^ question of the household *n*^th^, which supposedly receives only 0 or 1. *N*_*jt*_ represents the number of surveyed households in province *j* and also in year *t*. *N*_*q*_ is the number of questions described in Table 1.

**Fig 1:**
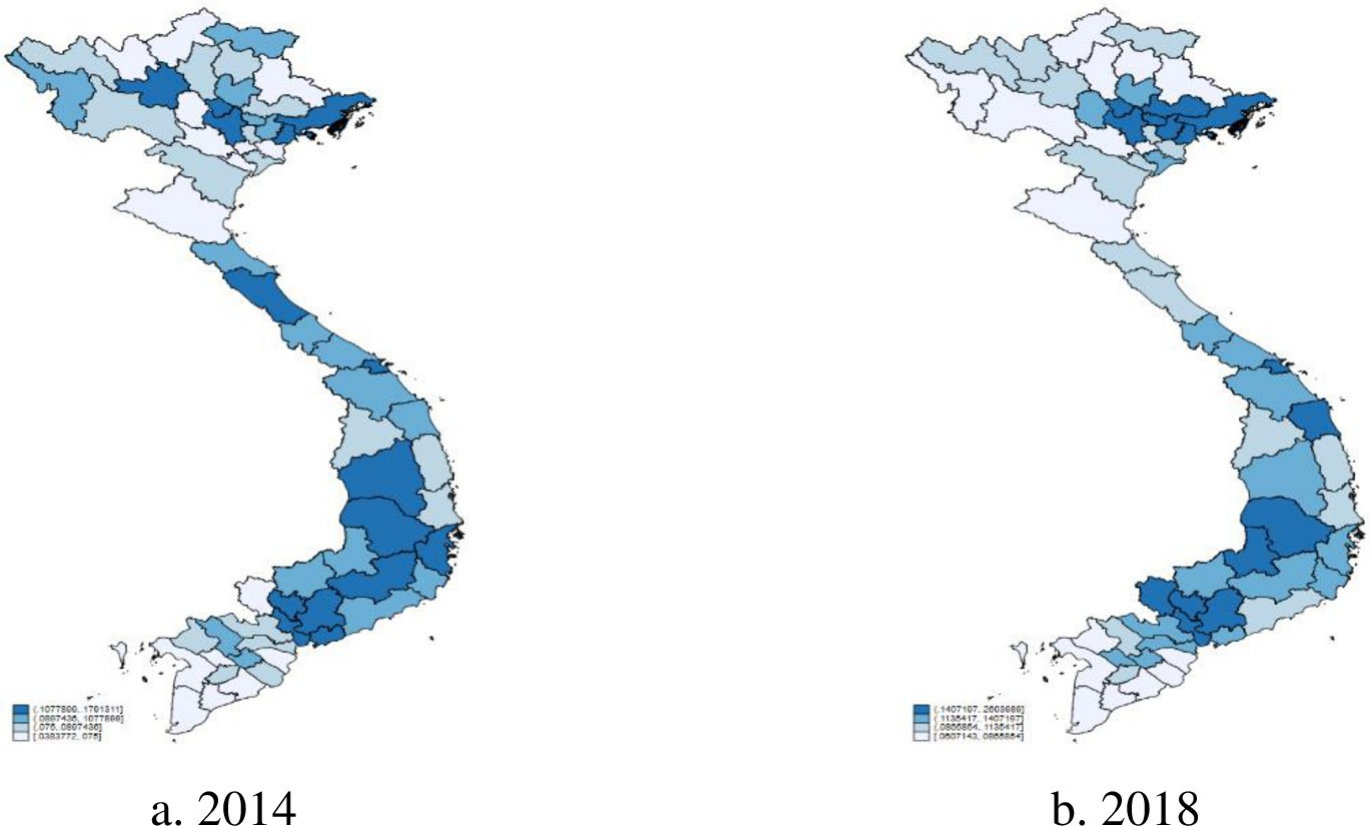
The financial inclusion index for 63 provinces in Vietnam.

Fig 1 exhibits the geographical dispersion of financial inclusion in Vietnam. As expected, the degree of financial inclusion proxied by financial usage appears to be higher in the provinces which belong to the Southern and Northern regions—Vietnam’s key economic regions. These two regions are currently the nation’s flagships for multiple high-tech and knowledge-based industries, manufacturing and services such as IT, telecommunication, electronics and software. Moreover, two national financial centres in Ho Chi Minh City and Hanoi capital city solely focus on developing the services sectors [65, 66]. We might also notice that the number of provinces, which are coloured using the boldest blue, experience a slight reduction. This visual evidence implies that Vietnam’s economic progression over the last four years reduces the proportion of provinces whose financial inclusion index belongs to the highest quartile and reallocates them into the nation’s two key economic regions. The spatial redistribution points to the increasing wave of migrants from rural to urban areas in Vietnam. Table 2 provides descriptive statistics of Vietnam’s provincial index of financial inclusion across years.

**Table 2:** The descriptive statistics of financial inclusion across Vietnam.

Year	Mean	SD	Min	Max
2014	0.093	0.030	0.038	0.179
2016	0.110	0.037	0.052	0.229
2018	0.120	0.042	0.061	0.260

As presented in Table 2, Vietnam’s economic growth does improve the overall level of our national financial inclusion, minimum and maximum. These findings imply that Vietnam’s economic growth and development is relatively inclusive.

We then rescale the range of our index of financial inclusion across provinces by taking the natural logarithmic transformation of the ratio $\frac{{IFI}_{it}}{\bar{IFI_{t}}}$. The logarithmic form is useful for data whose absolute difference between the minimum and the maximum is large. The transformation is expressed as follows.

$$RIFI_{it}=\ln\left(\frac{{IFI}_{it}}{\bar{IFI_{t}}}\right)=\ln\left(IFI_{it}\right)-\ln\left({\bar{IFI}}_{t}\right)$$

where *IFI*_*it*_ represents the index of financial inclusion of *i*^th^ province in year *t*. $\bar{IFI_{t}}$ is the national average financial inclusion in year *t*.

### 3.2. Methodology

#### 3.2.1. Spatial distribution of financial inclusion or the bivariate Kernel density estimation

In order to investigate the distribution of the degree of financial inclusion across provinces in Vietnam, we simulate the mass function of our index of financial inclusion using kernel density estimation. Many non-parametric methods can provide analysts with a profound understanding of the data distribution, such as the histogram, box plot, stem and leaf plot and cumulative distribution. However, the kernel smoothing density estimate is more efficient since it is neutral to the bad effects of arbitrary conventions or subdivisions [67]. Moreover, kernel estimation also considers the smooth transition from value to value based on the continuous scale. Doing so will optimally reduce the discreteness and the graphical deterioration given by the inclusion of outliers. In that sense, the kernel density function of the index of financial inclusion is estimated as below.

$$\hat{f}(x)=\frac{1}{N}{\det\left(h\right)}^{-1}\Sigma_{i=1}^{N}K[h^{-1}(x-x_{i})]$$ (1)

where *K* can be the Epanechnikov, Gaussian or Triangular distribution at the data point *x*_*it*_. The selection of kernel *K* expresses how well the data is centred around the realization *x*_*it*_. *h* represents the half-width (smoothing width or smoothing parameter), which determines each kernel’s relative width and height. *N* represents the number of realizations of a random variable *X*, which is the financial inclusion index. In sum, the probability density function *f*(*x*) of a random variable *X* is estimated by the sum of *N* identically distributed kernels placed on top of the data points.

**Fig 2:**
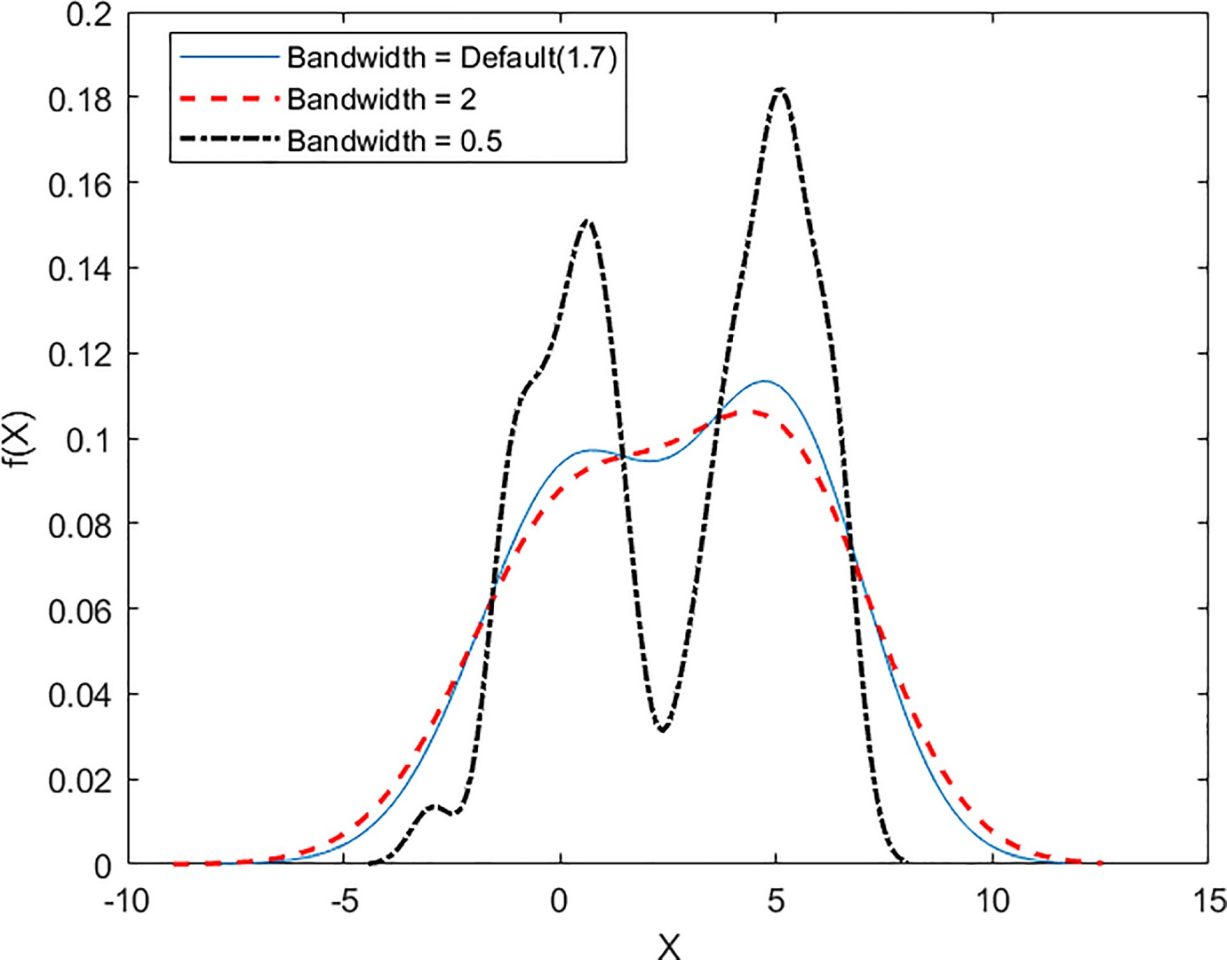
Graphical variation in bandwidths.

Based on Eq (1), we now consider the specification of the kernel *K*, which determines the juxtaposition and superimposition of the data and smoothing parameter *h*. It is widely acknowledged that the selection of *K* is less particularly crucial than the choice of *h* [68, 69] since the variation of kernel exerts the shape of the estimated mass function in practice [70]. However, the choice of half-width *h* is the primary practical concern since it heavily decides the discreteness of the estimated density function $\hat{f}(x)$. There are two problems derived from the selection of *h*, which are over and underestimate. Both of them are the two sides of the same coin. Literature on statistics considers the selected degree of smoothing as the trade-off problem. Under-smoothing occurs when the value of *h* is over-specified and vice versa for over-smoothing. We note that the excessively high value of *h* is associated with the discontinuity between observed data [71]. Fig 2 visualizes the importance of the selection of the bandwidth. The picture is drawn by applying the density estimation on the column vector that contains two pseudorandom algorithms. The smaller the reciprocal 1/*Nh* is, the pattern becomes unsmoothed. We also note that the pattern whose bandwidth is 0.5 will cluster into two spikes. The first spike is around 0, and the second spike is around 5, which is the actual means of the mixture of two pseudorandom normal distributions that we previously specified.

Fig 2 also indicates that slightly under-smoothing is commonly preferred over its counterpart since it retains the data’s underlying characteristics. This conclusion has been documented in the works of [67, 72]. Another solution for selecting the bandwidth is to allow the value of *h* to respectively change with the variation of *X* [73, 74]. The adaptive choice of *h* offers a more accurate estimation than the fixed method. Eq (1) is modified as below to integrate the variation of *h*:

$$\hat{f}(x)=\frac{1}{n}\sum_{i=1}^{n}\frac{1}{h_{i}}K(\frac{x-x_{i}}{h_{i}})$$ (2)

The last method optimizes the selection of *h* by minimizing the value of the MISE (the mean integrated squared errors). The term can be mathematically expressed as follows:

$$MISE(h)=E\left[\int {\left[{\hat{f}}_{h}(X)-f_{h}(X)\right]}^{2}dX\right]$$ (3)

where ${\hat{f}}_{h}(x)$ and *f*_*h*_(*x*) are respectively the estimated and the real distribution function of *X*. Given by Eq (3), the optimal value for *h* is $\alpha n^{-\frac{1}{5}}$ as the solution of the equation *h*′ = *argmin*{*MISE*(*h*)} where *α* is either *σ*_*x*_ or *IQR*_*X*_ (interquartile range) depending on which receives a smaller value. In this study, we utilize the last method.

#### 3.2.2. Dynamic distribution of financial inclusion/multivariate kernel density estimation

Analysis on economic convergence has increased incredibly since [7, 8] outlined the framework of accumulative capital. As they documented, developed countries gradually approach the steady-state of growth, where capital acts only as a share of output. Many scholars have attempted to validate and discover the condition of convergence, but the conclusion is still left unanswered since there is limited empirical evidence that supports the theory [14, 75–77]. Our study focuses on the methods that various scholars have previously employed to find the empirical evidence of convergence. Generally, there are two mainstreams, which are the parametric and non-parametric approaches. They are often used for empirically and mathematically depicting the identical concept of convergence. However, each one differently stipulates the requirements for achieving the state of convergence. The most widely applied method is the *β* convergence, whose name derives from the estimated coefficient of the dynamic setup between growth, initial income, human capital, physical capital and saving rates [78, 79]. In this method, the negative sign of the initial income and subsequent growth rate acts as a prerequisite for validating the theoretical framework. By doing so, a myriad of empirical studies provided diverse and controversial conclusions on the theory of convergence based on the difference in sample size and set of explanatory variables [78, 80, 81].

The *β* convergence itself also receives many criticisms since it oversimplifies the economic progression just by attributing it to the negative coefficient of the one-size-fits-all model [82, 83]. Debate that although the reduction in subsequent growth rate is needed for the convergence, it does not adequately capture the plummet in income dispersion. The idea inspires the emergence of the non-parametric approach of *σ*- convergence or the stochastic kernel density function, which describes the method of investigating the changes in the scatter of income [84, 85]. Likely to the *β*-convergence method, the *σ*-convergence is also deficient in explaining the dynamic structure, which lies underneath the distribution function of income over time. Given the deficiencies of conventional methods, we used the dynamic kernel density function, which proves to be efficient in visually capturing the catching-up effects and multimodality. However, few empirical attempts used this non-parametric approach to examine the underlying convergence of different economic phenomena [86]. Applies the dynamic kernel density function to examine the income inequality in the US from 1948 to 1993. Based on [87] analysis, [88] investigate the tendency of energy intensity in China [83]. Investigates the dynamic distribution of intangible capital in China. To the best of our knowledge, no attempt has been made using this method to examine the dynamics of financial inclusion in Vietnam.

Let denote the mass function of income at time *t* and *t*+*τ* respectively as *f*_*t*_(*X*) and *f*_*t*+*τ*_(*Z*), where *τ* is the positive integer *τ*∈*N*. We also assume that the tendency of using financial services is a function of the financial literacy, income, cultural and ideological characteristics [89, 90], which are likely held constant over time except for income. Then, the mass function *f*_*t*+*τ*_(*Z*) at time *t*+*τ* is expressed as:

$$f_{t+\tau }(Z)=\int_{-\infty }^{+\infty }f_{\tau }(Z|x)f(X)dx$$

Where *f*_*τ*_(*Z*|*x*) is the conditional density function that represents the dependent structure of IFI at different points in time. In other words, the conditional density function maps the value of IFI of any province in time *t* to its IFI at time *t*+*τ*. Since *f*_*τ*_(*Z*|*x*) is the conditional distribution function, it must satisfy: $\int_{-\infty }^{+\infty }f_{\tau }(Z|x)dz=1,\forall x\in X$, where *X* is the vector of random variable IFI and *x* is the realization of *X*. Let ${\hat{f}}_{t,t+\tau }(Z,X)$ is the estimated kernel density function of *X* and *Z*, which is the dynamic mass function of *X* at time *t* and *t*+*τ*. Then, *f*_*τ*_(*Z*|*x*) is defined as $f_{\tau }(Z|x)=\frac{{\hat{f}}_{t,t+\tau }(X,Z)}{\hat{f_{t}}(X)}$, where the denominator is the kernel density estimate of *X* at time *t*. As *τ* approaches infinity, *f*_∞_(*Z*) also approaches infinity, which gives us the ergodic distribution of *Z* as follow.


$$f_{\infty }(Z)=\int_{0}^{\infty }f_{\tau }(Z|x)f_{\infty }(X)dx$$

Similar to the bivariate kernel density estimation, the ${\hat{f}}_{t,t+\tau }(Z,X)$ is computed as follow:

$${\hat{f}}_{t,t+\tau }(Z,X)=\frac{1}{Nh_{z}}\frac{1}{h_{x}}\sum_{i=1}^{N}K\left[\frac{z-z_{i}}{h_{z}},\frac{x-x_{i}}{h_{x}}\right]$$ (4)

Where *N* is the number of provinces in the sample, *h*_*z*_ and *h*_*x*_ are respectively the optimal bandwidths for vectors of random variable *Z* and *X*, whose *z*_*i*_ and *x*_*i*_ are their realizations. *K* in Eq (4) denotes either Epanechnikov, Gaussian, Cosine or Triangular kernel density function.

As [88, 91] mentioned, we are occasionally interested in knowing the probability of ascending to the higher state of an investigated subject against its descending one. This is the case where the mobility probability plot (MPP) exhibits its usefulness. MPP advantageously facilitates the comprehension of results by offering a net probability at one particular point, which is derived by taking the probability of moving up at a particular point *x* = *x*_*i*_ and then subtracting it to move down. It is more often favourable to contour map plots, 3D-map plots and ergodic distribution. The calculating process is mathematically expressed as:

$$p(x)=\int_{x}^{+\infty }f_{\tau }(Z|x)dZ-\int_{-\infty }^{x}f_{\tau }(Z|x)dZ$$

As mentioned above, the *f*_*τ*_(*Z*|*x*) depends on the estimated distribution function of ${\hat{f}}_{t}(x)$, whose values heavily depend on the sample’s representativeness. Moreover, the estimated mass function ${\hat{f}}_{t}(x)$, which is symbolic of the provincial level of financial inclusion of Vietnam in year *t*, heavily depends on the sampling process. Technically, we have to create an estimated range for the unknown population *f*_*t*_(*x*) to bounce. Therefore, we follow the five-step procedure of creating the confidence interval (CI) for unknown MPP, as [83] documented. First, we randomly draw with replacement (bootstrapping) from the column vector of random variable IFI *n* = 63×2 values. Then we label this series as *X*. Second, we locate the one period after values of *X* and stack them into one column vector and then call it as *Z*. Third, create the matrix of *Z*|*X*. Fourth, using *X* and *Z*|*X* to estimate the multivariate density function ${\hat{f}}_{t,t+\tau }(Z,\;X)$ described in the Eq (4), draw out the 3D and contour map. Fifth, compute the *p*(*x*) and repeat the entire process 1,000 times.

With that construction of CI, we could empirically ascertain two things. First, we can assure the likelihood that the regional index of financial inclusion (RIFI) of a particular province likely to enhance or degrade given by Vietnam economic progression is significantly different from zero or not. Second, we can also assure the tendency of the financial inclusion in Vietnam to likely decrease or increase in the future.

## 4. Empirical findings

### 4.1. Static analysis

**Fig 3:**
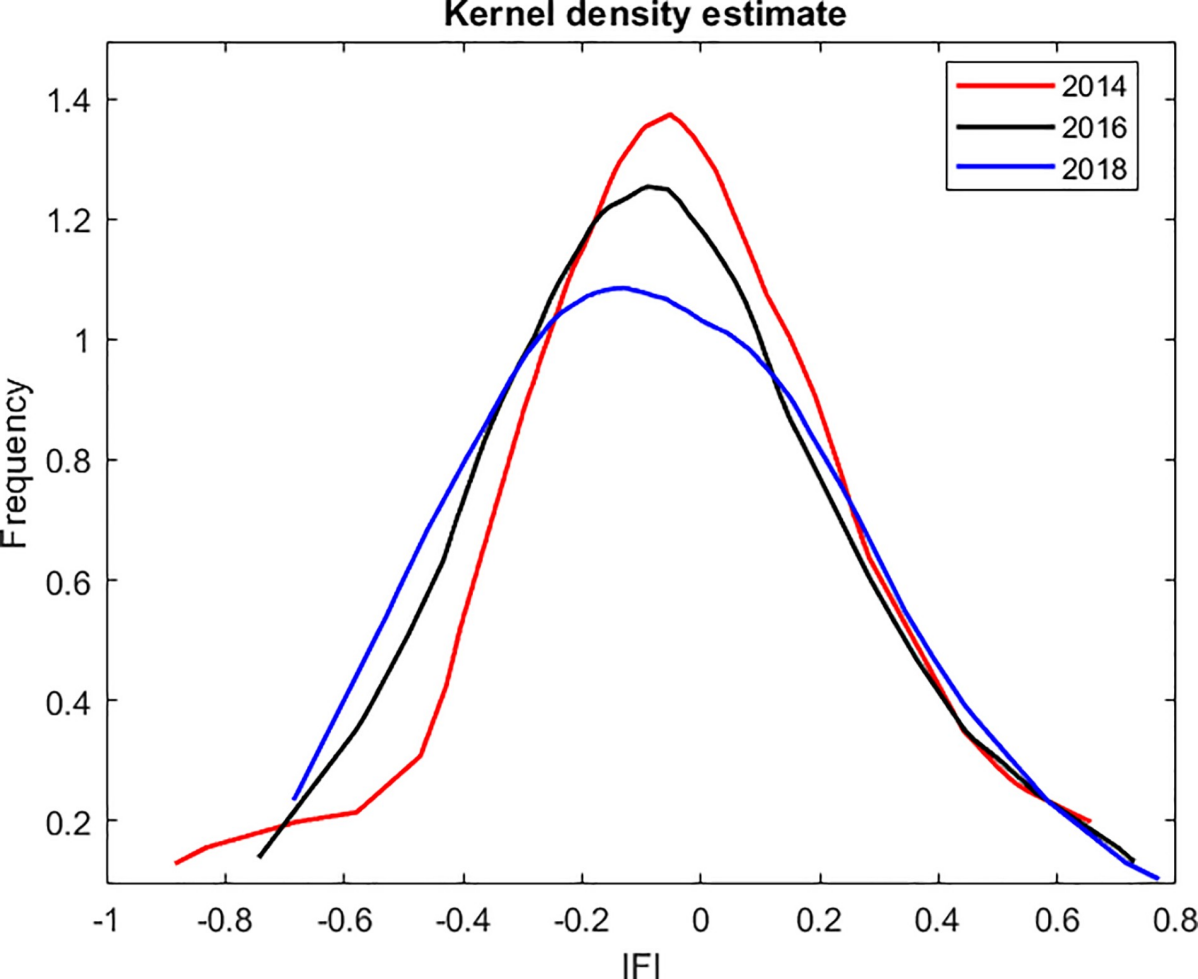
The distribution of the regional index of financial inclusion (RIFI).

Fig 3 below represent the distribution function of the RIFI by year. The peaks of the three functions are around zero and backward shifting. This finding implies that most provinces whose people with access to finance at the national average exhibit a decreasing tendency. The left-hand side of the bell-shaped curve in 2014 is steeper than those in 2016 and 2018. This finding means that the number of provinces with low RIFI has increased. As presented in Table 2, the proportion of people with access to financial services and instruments is high only in developed provinces such as Ho Chi Minh City and Hanoi, which also belong to the key economic regions. The left and right tails of the distribution of the RIFI in 2014 slightly shift backwards, which means that economic growth enhances the financial accessibility of both left-tailed and right-tailed provinces. We also observe the sign of bimodality since the peaks of those distributions are flattened by year.

### 4.2. Dynamic analysis

#### 4.2.1. Intra-distribution mobility

**Fig 4:**
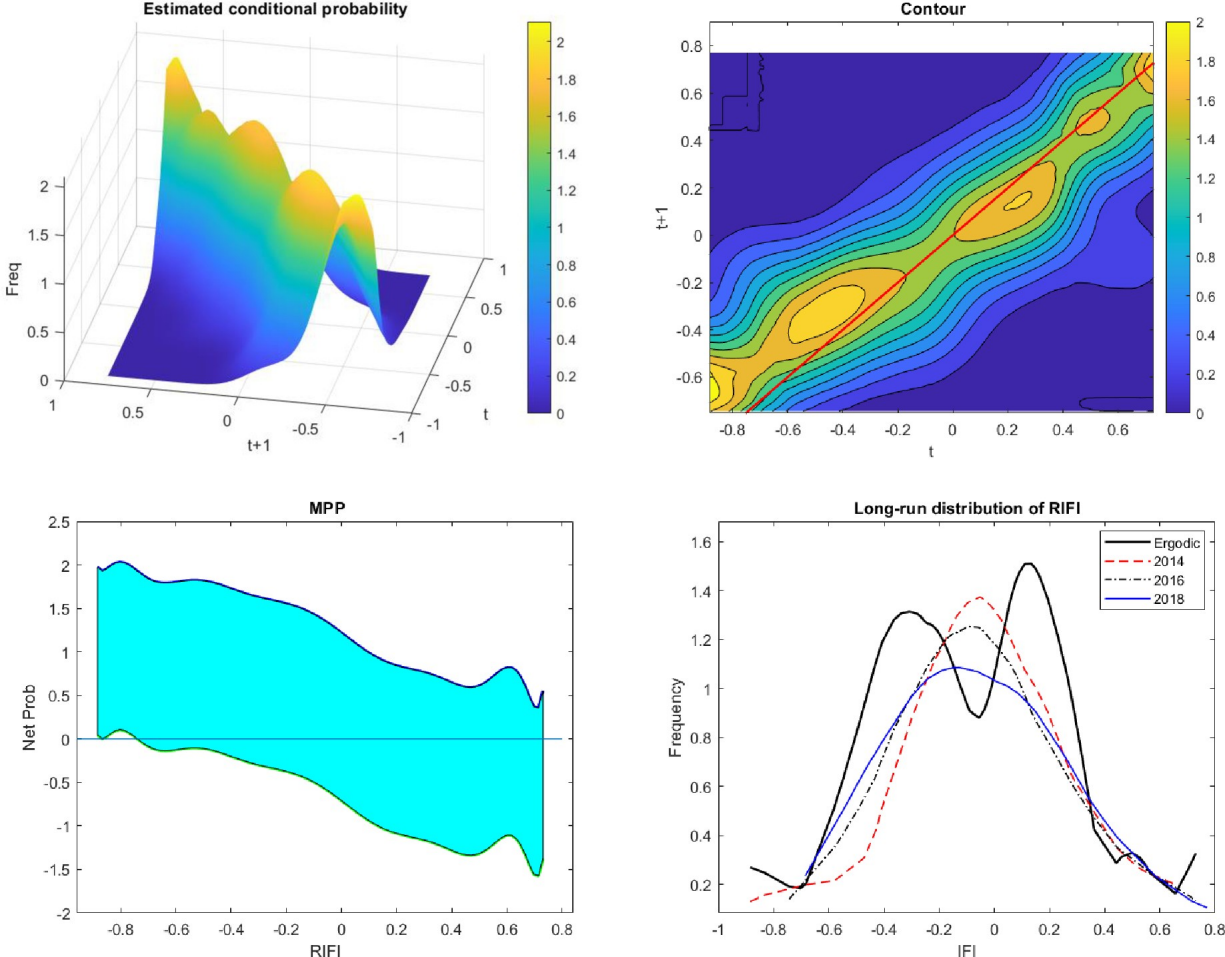
The transitory distribution of financial inclusion in Vietnam.

Fig 4 demonstrates the dynamic analysis of the distribution function of 63 provinces in Vietnam over the 2014–2018 period. The 3D surface and contour visualize the transition in a mathematical form of a conditional probability. These are probabilities of a province receiving different possible values of RIFI after a year, conditioned by the current value at time *t*. In other words, the 3D surface is the alignment of the infinite RIFI density function at time *t*+1, given by the value at time *t*. The contour is just the projection of the first sub-figure onto the 2D dimension. The third sub-figure provides explanations for the net probability of moving up or down at any specific value in time *t*. As presented in the contour, provinces whose RIFI are in the first quartile are most likely to experience the uplift since the peak corresponds to the below-average part, which appears to lie above the 45-degree line.

Similarly, provinces with an initially high degree of financial inclusion likely remain unchanged in the next year since the 45 degree runs over the peak of their transitive distribution. This finding implies that provinces whose financial usages are relatively lower than the national average exhibit the increasing tendency of financial inclusion during the period 2014–2018. The ergodic function is presented in the fourth sub-figure, which implies the long-run bimodality given by the current state of economic growth and development. In other words, the development, in the long run, categorizes Vietnam’s provinces into two groups: (i) the so-called “the financially familiar group” and (ii) “the financially unfamiliar group”. The group of provinces whose economic growth is more financially focused experiences a higher and steadier growth than the remaining group. Moreover, the financially familiar group includes provinces that belong to the two key economic regions. In these provinces, a level of financial inclusion is significantly higher than that of other provinces outside the two key economic zones.

The degree of financial inclusion obtained from these financially advantaged provinces within the two key economic regions appears to converge during the 2014–2018 research period. For provinces outside the two key economic zones, the geographically disadvantaged provinces mainly participate in the agricultural sector. However, the contour and the mobility probability plot (MPP) graph exhibit the persistent tendency of financial inclusion in the short run because the majority fluctuates around the zero points. In addition to the persistent tendency observed along the x-axis in the MPP graph, provinces whose RIFI varies within the [-0.8, -0.2] interval are more likely to experience an upward transition after a year.

The ergodic function only gives us a glance at the future distribution of RIFI. As such, we have no evidence on how and to what extent RIFI changes dynamically within the two groups. In this general transitive analysis, we assume that provinces within the two key economic regions have the advantage of increasing the number of people with access to the financial sector.

#### 4.2.2. Spatial dynamic distribution

**Fig 5:**
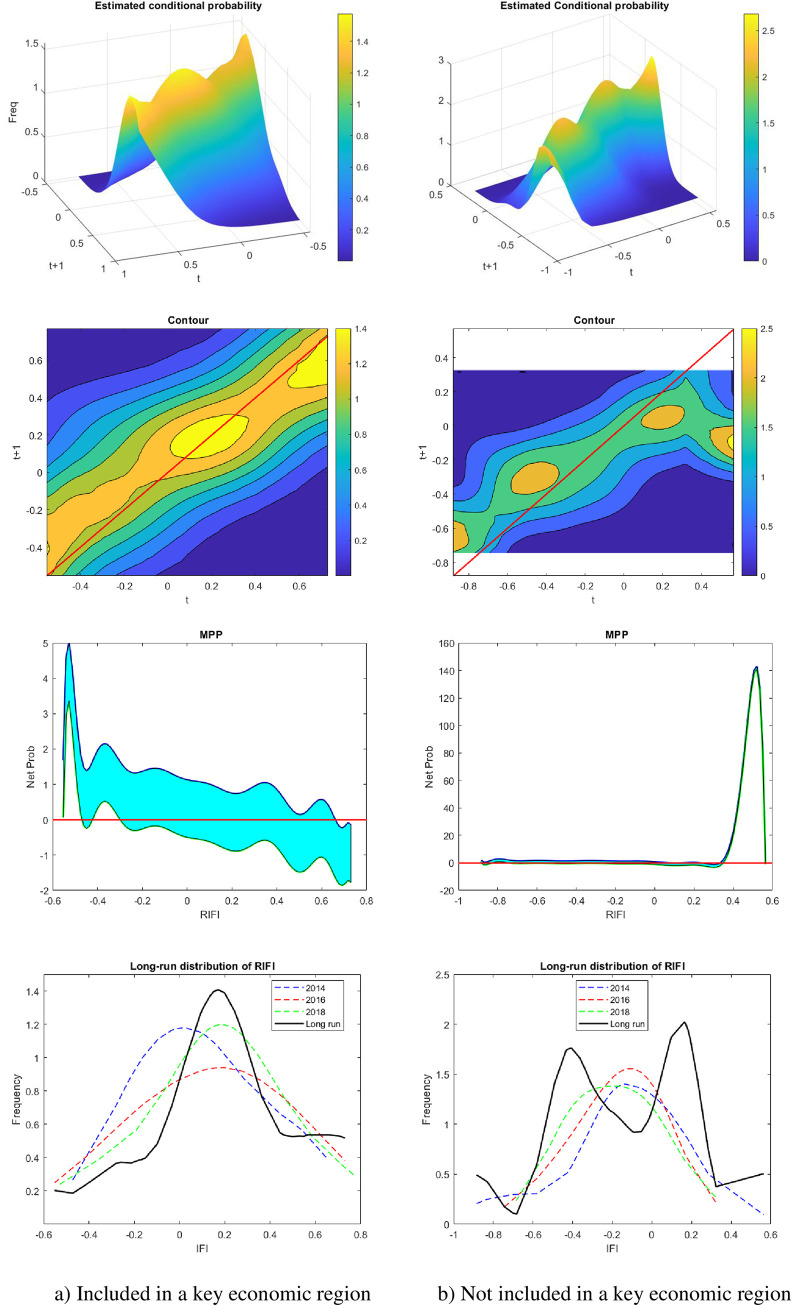
Financial inclusion of two groups of provinces.

In order to provide insightful analysis on the RIFI distribution, we divide our sample into two groups as suggested by the results from the previous ergodic function. The first group includes provinces that belong to the key economic regions. The provinces in the key economic zones are established according to the 15/2008/QH12 Resolution enacted by the Vietnam Congress on 15 August 2008. The second group comprises the remaining provinces in Vietnam. Using the same process as described in section 3, Fig 5 below provides a clear progression of financial inclusion across provinces in Vietnam. Evidence from Fig 5 confirms that both groups of provinces exhibit the convergence of financial inclusion. With those included in the two key economic zones, the level of financial inclusion is higher than the national average and tends to cluster around the point of 0.2. This is illustrated by the hotter colour in the contour and the uncertainty of mobility in the MPP at the point of 0.2. For provinces located outside two key economic regions, the range varies from -0.4 and 0.2. These findings imply that provinces, which are not considered national flagships of advanced manufacturing and services, exhibit the convergence of the RIFI among provinces within the same region and catch up with other provinces. Some provinces within this group might achieve the same level of financial inclusion as provinces of the financially focused group in the long run. However, a bimodal structure lies underneath the dynamic distribution of the RIFI in these provinces, which are not located in the two economic regions. Noticeably, the disparity of the RIFI happens between the left tailed and right-tailed provinces, which explains the bimodality of a national long-run ergodic function presented in Fig 4. In other words, financial usage across Vietnamese provinces exerts both convergence and divergence. Despite the disparity in the long run distribution, financial inclusion of the Vietnamese provinces, as shown in Figs 4 and 5, is noticeably improved, especially for provinces, within key economic regions located on the left tail of the distribution and the right tail of the other regions. The peak of the conditional probability of the left-tailed provinces lies above the 45-degree line, indicating a strong tendency for improvement in these provinces. In other words, the government’s programs designed to improve the usage of financial services, to some extent, do help narrow the gap between provinces with a low and high proportion of people with access to financial amenities.

Our empirical findings align with [25] findings, [26] studies. These studies provide evidence to confirm that locations with advantaged conditions will be likely to achieve higher economic performance and tend to lead the disadvantaged ones concerning the degree of financial inclusion. In other words, these country-wise economic asymmetries or, in our study, provincial differences in the access to finance can be explained by the dissimilarity in the initial wealth, which includes human capital and physical capital stock.

In summary, with the commencement of multiple pro-poor programs initiated and implemented by the government since 2010, many of these programs prove to be efficient in reducing the number of households living in poverty by making progress in their accessibility to various financial amenities. Vietnam’s current strategies of financial inclusion demonstrate simultaneous divergence and convergence. Residents in the financially advantaged provinces are financially proficient. The gap between access to financial services between the financially advantaged provinces and the financially disadvantaged provinces is gradually widened.

## 5. Conclusions

Literature shares consensus about the contribution of finance to growth. As such, low financial accessibility is generally considered suboptimal to support economic growth. Although the term “financial inclusion” was introduced in the early 20^th^ century, Vietnam and other emerging countries have only considered and adopted critical views on this matter in the last decade. For Vietnam, it has been a decade since the general statistics office (GSO) started monitoring how Vietnamese households at different administrative units are familiar with the financial sector by incorporating and asking questions on the financial status. Despite commendable improvements, such as the increase in the number of hamlets that have commercial bank branches [10], Vietnam has been struggling to promote financial accessibility and literacy. We find that Vietnam’s long-run pattern of financial access contains bimodality, which means that the gap between financially advantaged and disadvantaged provinces is widened over time.

To an end, policy implications have emerged based on the findings from our analysis. *First*, the growth of a non-agricultural sector has caused the waves of emigration from rural to urban areas in seeking work opportunities. In addition, the rapid expansion of industries, which generate substantial cash flows from operations, exerts environmental pressure and causes provincial disparity in economic growth and financial accessibility. As such, the Vietnamese government should consider adopting policies that aid the modernization of the agricultural sector and, as a consequence, improve the productivity of the sector. *Second*, creating an effective lending mechanism that generates accustomed services and reducing the information asymmetry between lenders and borrowers is of utmost importance for traditional and unbanked farmers who withstand the idiosyncratic shocks from natural disasters or efficiently improve their traditional approach to production.

## Supporting information

S1 File (RAR)
